# Assessment of three Resistance-Nodulation-Cell Division drug efflux transporters of *Burkholderia cenocepacia *in intrinsic antibiotic resistance

**DOI:** 10.1186/1471-2180-9-200

**Published:** 2009-09-17

**Authors:** Silvia Buroni, Maria R Pasca, Ronald S Flannagan, Silvia Bazzini, Anna Milano, Iris Bertani, Vittorio Venturi, Miguel A Valvano, Giovanna Riccardi

**Affiliations:** 1 Department of Genetics and Microbiology, University of Pavia, 27100 Pavia, Italy; 2 Infectious Diseases Research Group, Department of Microbiology and Immunology, Siebens-Drake Research Institute, University of Western Ontario, London, ON N6A 5C1, Canada; 3 Bacteriology Group, International Center for Genetic Engineering & Biotechnology, Padriciano 99, 34012 Trieste, Italy

## Abstract

**Background:**

*Burkholderia cenocepacia *are opportunistic Gram-negative bacteria that can cause chronic pulmonary infections in patients with cystic fibrosis. These bacteria demonstrate a high-level of intrinsic antibiotic resistance to most clinically useful antibiotics complicating treatment. We previously identified 14 genes encoding putative Resistance-Nodulation-Cell Division (RND) efflux pumps in the genome of *B. cenocepacia *J2315, but the contribution of these pumps to the intrinsic drug resistance of this bacterium remains unclear.

**Results:**

To investigate the contribution of efflux pumps to intrinsic drug resistance of *B. cenocepacia *J2315, we deleted 3 operons encoding the putative RND transporters RND-1, RND-3, and RND-4 containing the genes *BCAS0591*-*BCAS0593*, *BCAL1674*-*BCAL1676*, and *BCAL2822*-*BCAL2820*. Each deletion included the genes encoding the RND transporter itself and those encoding predicted periplasmic proteins and outer membrane pores. In addition, the deletion of *rnd-3 *also included *BCAL1672*, encoding a putative TetR regulator. The *B. cenocepacia rnd-3 *and *rnd-4 *mutants demonstrated increased sensitivity to inhibitory compounds, suggesting an involvement of these proteins in drug resistance. Moreover, the *rnd-3 *and *rnd-4 *mutants demonstrated reduced accumulation of N-acyl homoserine lactones in the growth medium. In contrast, deletion of the *rnd-1 *operon had no detectable phenotypes under the conditions assayed.

**Conclusion:**

Two of the three inactivated RND efflux pumps in *B. cenocepacia *J2315 contribute to the high level of intrinsic resistance of this strain to some antibiotics and other inhibitory compounds. Furthermore, these efflux systems also mediate accumulation in the growth medium of quorum sensing molecules that have been shown to contribute to infection. A systematic study of RND efflux systems in *B. cenocepacia *is required to provide a full picture of intrinsic antibiotic resistance in this opportunistic bacterium.

## Background

*Burkholderia cenocepacia *is a member of the *Burkholderia cepacia *complex (Bcc), a group of phenotypically similar Gram-negative bacteria [1] that are opportunistic pathogens and sometimes cause serious life-threatening infections in cystic fibrosis (CF) patients [2,3]. Infection in CF patients may result in asymptomatic carriage, but often leads to a rapid decline of the lung function and in some cases to the "cepacia syndrome", characterized by necrotizing pneumonia and sepsis [4].

*B. cenocepacia *and other members of the Bcc demonstrate high-levels of intrinsic resistance to most clinically relevant antibiotics, complicating the treatment of the infection [5]. Multi-drug resistance in CF isolates is defined as resistance to all of the agents in two of three classes of antibiotics, such as quinolones, aminoglycosides, and β-lactam agents, including monobactams and carbapenems [6]. Multiple antibiotic resistances in Bcc bacteria have been attributed to reduced permeability of the bacterial outer membrane [7-9], expression of antibiotic modifying enzymes [10], and alteration of cellular targets [11]. Information relating to the contribution that drug efflux systems play in the drug resistance of Bcc bacteria is limited, as only a few multi-drug efflux pumps have been described to date in some clinical isolates [12-14]. In contrast, the contribution of multidrug efflux systems to antibiotic resistance in clinical isolates of *Pseudomonas aeruginosa*, another CF pathogen, is well documented. Two *P. aeruginosa *efflux pumps, MexAB-OprM and MexXY-OprM, contribute to intrinsic multidrug resistance, while MexCD-OprJ and MexEF-OprN are responsible for the acquired antimicrobial resistance of different mutant strains [15].

RND transporters are important mediators of multi-drug resistance in Gram-negative bacteria [16]. RND transporters form protein complexes that span both the cytoplasmic and outer membrane. The complex comprises a cytoplasmic membrane transporter protein, a periplasmic-exposed membrane adaptor protein, and an outer-membrane channel protein. The *Escherichia coli *AcrAB-TolC and the *P. aeruginosa *MexAB-OprM complexes are extremely well characterized and the three-dimensional structures of various components have been resolved [17-21].

Two RND type multi-drug efflux pumps, AmrAB-OprA and BpeAB-OprB, have been described in *Burkholderia pseudomallei *(the causative agent of melioidosis) and both confer resistance to aminoglycosides and macrolides [22,23]. The contribution of BpeAB-OprB and AmrAB-OprA, to the intrinsic resistance of *B. pseudomallei *to gentamicin, streptomycin and erythromycin explains why aminoglycoside-β-lactam combinations, which are commonly used to treat suspected cases of community-acquired sepsis in any part of the world, are ineffective for the treatment of melioidosis [24]. Furthermore, the transport of acyl homoserine lactones, involved in quorum-sensing systems of *B. pseudomallei*, also requires the BpeAB-OprB efflux pump [25]. Thus, targeted inhibition of BpeAB-OprB could be therapeutically beneficial.

Several possible strategies can be considered to specifically block the activity of these drug efflux pumps, such as jamming the outer membrane channel, generating competition at the inner membrane pump, altering the pump assembling or collapsing the energy component of the mechanism [26]. The activity of efflux inhibitors, such as diamine compounds, has been demonstrated in animal models of *P. aeruginosa *infections and two of them are in preclinical development [26].

In *B. cenocepacia *the significance of RND efflux systems has not been determined. However, a salicylate-regulated efflux pump that is conserved among members of the Bcc has been identified [27,28]. We are focusing our research in the *B. cenocepacia *J2315 strain. This strain is a prototypic isolate belonging to an epidemic clone that has spread by cross infection to CF patients in Europe and North America [29]. Previously, we identified 14 genes encoding putative RND efflux pumps in the genome of *B. cenocepacia *J2315 [30]. After the completion of the whole genome sequence [31], two additional genes encoding RND pumps were discovered. Reverse transcriptase analyses showed that some of these genes are indeed transcribed at detectable levels. As a first step towards understanding the contribution of RND pumps to *B. cenocepacia *antibiotic resistance we deleted genes encoding putative efflux pumps, RND-1, RND-3, and RND-4, containing the genes *BCAS0591*-*BCAS0593 *(located on chromosome 3), *BCAL1674*-*BCAL1676*, and *BCAL2822*-*BCAL2820 *(located on chromosome 1), respectively. In a previous publication, the genes encoding the membrane transporter component of the efflux pump, BCAS0592, BCAL1675, and BCAL2821 were referred to as Orf1, Orf3, and Orf4, respectively [30]. In this investigation we show that deletion of *rnd-3 *and *rnd-4 *genes is associated with increased sensitivity to certain antibiotics and reduced secretion of quorum sensing molecules.

## Results and Discussion

### *B. cenocepacia *BCAS0592, BCAL1675, and BCAL2821 encode RND-type transporters

**Figure 1:**
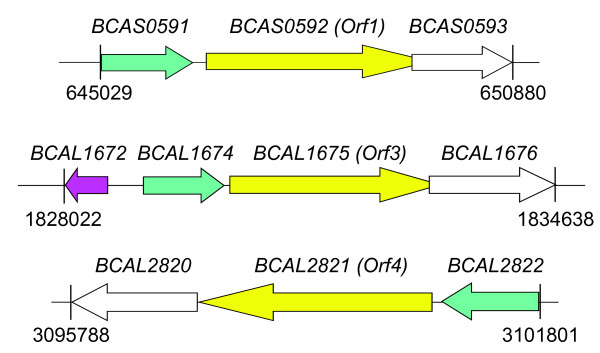
**Genetic map of *B. cenocepacia rnd *operons containing the *BCAS0592*, *BCAL1675*, and *BCAL2821 *genes**. Gene positions and orientations are shown. Membrane fusion protein encoding genes are depicted in green, the RND encoding ones in yellow (the previous name attributed to these genes in reported in parentheses), and the genes encoding outer membrane proteins are in white. The putative repressor gene *BCAL1672 *is depicted in pink.

We characterized 3 efflux systems of *B. cenocepacia *J2315 by deletion mutagenesis. These systems were selected based on their high homology to the well-characterized Mex efflux pumps in *P. aeruginosa*. One of the identified operons, located on chromosome 3, encodes RND-1 and comprises the genes *BCAS0591-BCAS0592-BCAS0593 *that span nucleotides 645029 to 650880 [Fig. 1]. *BCAS0591 *encodes a predicted 418-aa membrane fusion protein, followed by the RND transporter gene predicted to encode a 1065-aa protein, and *BCAS0593 *encoding a 475-aa outer membrane protein. Amino acid sequence analysis of the *BCAS0592 *gene product revealed conserved motifs and the characteristic predicted structure common to the inner membrane proteins of the RND efflux complex. Topologically BCAS0592 is a polypeptide with 12 predicted transmembrane alpha helices and two large periplasmic loops between transmembrane helices 1-2 and 7-8 [30]. A search using the National Center for Biotechnology Information protein database with the BLASTP program http://www.ncbi.nlm.nih.gov/ revealed that the components of this efflux system shared amino acid sequence identity with the well characterized AcrAB-TolC, BpeAB-OprB, and MexAB-OprM RND efflux pumps of *E. coli*, *B. pseudomallei*, and *P. aeruginosa*, respectively. In particular, BCAS0592 shared 60, 59, 56% amino acid identity with the RND transporters AcrB (*E. coli*), BpeB (*B. pseudomallei*), and MexB (*P. aeruginosa*), respectively. BCAS0591 shared 53, 50, and 50% amino acid identity with the membrane fusion proteins AcrA (*E. coli*), MexA (*P. aeruginosa*), and BpeA (*B. pseudomallei*). On the other hand, BCAS0593 shared 52% amino acid identity with OprM (*P. aeruginosa*) and 49% with OprB (*B. pseudomallei*), both of which are outer membrane pore proteins.

The operon encoding RND-3 is located on chromosome 1 and spans nucleotides 1830038 to 1834638. The first gene, *BCAL1674*, encodes the membrane fusion protein, a predicted 406-aa protein. The product of the downstream gene is a predicted 1046-aa protein that functions as an RND transporter. The third gene, *BCAL1676*, encodes the 486-aa outer membrane pore protein [Fig. 1]. BLASTP results revealed that BCAL1674 had 79 and 48% identity with the membrane fusion proteins AmrA (*B. pseudomallei*) and MexC (*P. aeruginosa*), while BCAL1675 was similar to AmrB (*B. pseudomallei*, 86%) and to MexD of *P. aeruginosa *(52%), both encoding the RND transporter. BCAL1676 was highly related to the outer membrane proteins OprA of *B. pseudomallei *(78% of identity) and OprM of *P. aeruginosa *(47%) and again possessed the predicted conserved structural features of outer membrane proteins that function in RND efflux systems. A gene encoding a predicted TetR family regulator protein (*BCAL1672*) is located upstream of *BCAL1674 *but is transcribed in the opposite direction [Fig. 1].

Lastly, the predicted operon encoding RND-4, comprising the genes *BCAL2820*, *BCAL2821 *and *BCAL2822*, is located on chromosome 1 and spans nucleotides 3095788 to 3101801 [Fig. 1]. *BCAL2821 *encodes the 1066-aa RND transporter protein, which is highly related to BpeB from *B. pseudomallei *(94% identity) and to MexB (*P. aeruginosa*, 64% identity). *BCAL2820 *encodes the 507-aa outer membrane protein related to OprB (*B. pseudomallei*, 84% identity) and to OprM from *P. aeruginosa *(53% identity). *BCAL2822 *encodes a predicted 424-aa membrane fusion protein highly similar to BpeA from *B. pseudomallei *(89% identity) and to MexA from *P. aeruginosa *(54% identity).

To demonstrate a role for these efflux pumps in the intrinsic antibiotic resistance of *B. cenocepacia *J2315, we attempted the construction of single deletion mutants of each *rnd *gene using the method described by Flannagan *et al*. [32] (see Methods). The deletion mutagenesis strategy requires expression of the endonuclease I-*Sce*I and allows for the creation of unmarked gene deletions. While attempting to generate the deletion mutants we encountered difficulties selecting recombinant colonies at a high concentration of antibiotics. Similarly we also failed to identify positive colonies having targeted integration of the deletion plasmid. The latter was particularly difficult for our initial attempts to get single deletions of each of the *rnd *genes. We reasoned that the flanking regions of the *rnd *genes, which are cloned into the mutagenesis plasmid pGPI-*Sce*I to mediate targeted integration into the chromosome, share significant sequence identity between different *rnd *genes throughout the *B. cenocepacia *genome. Due to these difficulties we concluded that single gene deletions could not be possible using the I-*Sce*I mutagenesis strategy. To circumvent this problem we generated plasmids designed to delete the entire operons encoding the three different efflux systems, as the DNA flanking the operons was not similar between different operons encoding efflux systems. This strategy resulted in the mutant strains D1 (Δ*BCAS0591-BCAS0593*), D3 (Δ*BCAL1672-BCAL1676*), and D4 (Δ*BCAL2820-BCAL2822*). In the case of strain D3, the deletion not only included the *rnd *operon but also *BCAL1672*, encoding a putative TetR regulator. The presence of the correct deletion in each strain was confirmed by PCR analysis and Southern blot hybridization (data not shown).

### Effect of deletion of efflux pumps operons on *B. cenocepacia *J2315 drug resistance

To determine if the deletion of the targeted efflux pumps altered susceptibility to antimicrobial agents we exposed the parental strain J2315 and the mutants D1, D3, and D4 to a variety of antimicrobial compounds. Table 1 summarizes the minimum inhibitory concentrations (MICs) of the different compounds tested. The wild-type strain, J2315, demonstrates a high intrinsic level of resistance to a variety of drugs including β-lactams, aminoglycosides, fluoroquinolones, and ethidium bromide. Strain D1 (Δ*BCAS0591-BCAS0593*) did not show any increased susceptibility as compared to the parental strain J2315. The inability to demonstrate growth inhibition of *B. cenocepacia *D1 is likely due to functional redundancy as this strain carries genes encoding 15 other RND efflux pumps that could compensate for deletion of the *rnd-1 *operon. On the other hand, not all the RND efflux pumps seem to share the same drug specificity, and the selected compounds could be extruded from the cell by other transporters of non-RND families. In contrast, the deletion of the complete *BCAL1674-BCAL1675-BCAL1676 *operon (encoding the RND-3 efflux pump) and the *BCAL1672 *gene, encoding a putative transcriptional regulator, led to a 8-fold reduction in the MIC values for nalidixic acid as compared to the control strain J2315 [Table 1]. This result demonstrates that RND-3 is indeed required for antibiotic resistance and that, at least for the compounds tested, demonstrates nalidixic acid specificity as this was the only MIC altered in the mutant strain.

**Table 1:** Antimicrobial susceptibilities of *B. cenocepacia *J2315, D3, and D4 strains

Compound	MIC (μg/ml)		
	**J2315 wt**	**D3**	**D4**

Aztreonam	2000	2000	250
Ethidium bromide	>2000	>2000	125
Chloramphenicol	4	4	<1
Gentamicin	>2000	>2000	1000
Tobramicin	1000	1000	250
Nalidixic acid	16	2	4
Ciprofloxacin	8	8	2
Levofloxacin	4	4	0.5
Norfloxacin	32	32	8
Sparfloxacin	8	8	1

As already mentioned, the proteins BCAL1674, BCAL1675, and BCAL1676 that comprise the *rnd*-3 operon share strong sequence similarity to RND efflux pump AmrAB-OprA from *B. pseudomallei *which is responsible for the efflux of aminoglycosides and macrolides in that *Burkholderia *species [33]. We previously showed that the gene encoding the pump protein (*orf3*) was expressed at detectable levels by RT-PCR. Assuming that RND-3 is functionally similar to AmrAB-OprA, the lack of aminoglycoside and macrolide resistance in the *B. cenocepacia *D3 mutant may be due to an alternative efflux pump or resistance mechanism against aminoglycosides and macrolides. To address the notion of RND efflux pump redundancy, we are in the process of generating a complete library of RND deletion mutants that can be screened for drug sensitivity. Furthermore the I-*Sce*I deletion strategy makes it possible the construction of strains carrying multiple RND gene deletions, which we are also pursuing.

The *B. cenocepacia *D4 deletion mutant demonstrated a 4 to 16-fold increase in drug susceptibility to several of the antimicrobials tested, indicating that RND-4 plays an important role in the intrinsic antibiotic resistance of *B. cenocepacia *[Table 1]. In particular, strain D4 is more susceptible than the parental strain J2315 when exposed to aztreonam, chloramphenicol, gentamicin, tobramicin, and to different fluoroquinolones, such as nalidixic acid, ciprofloxacin, levofloxacin, norfloxacin, and sparfloxacin. Furthermore, the MIC of ethidium bromide was more than 16-fold lower in D4 than in J2315 [Table 1]. The MIC values for other drugs such as ampicillin, ceftazidime, meropenem, piperacillin, erythromycin, and kanamycin were not altered in D4 as compared to J2315 (data not shown). Increased sensitivity to many antimicrobials of therapeutic importance might suggest that inhibition of RND-4 function could be of benefit to CF patients colonized with *B. cenocepacia*.

### Effect of broad-spectrum efflux pump inhibitor MC-207,110 on *B. cenocepacia *J2315 and the RND deletion mutants D1, D3 and D4

It has been reported that MC-207,110 efflux inhibitor has a potentiating effect in *P. aeruginosa*, where it lowers the MIC of different fluoroquinolones [34,35]. We tested the effect of this efflux inhibitor on *B. cenocepacia *J2315 and the deletion mutants D1, D3, and D4. To initiate this analysis we determined the MIC of MC-207,110 for our bacterial strains to determine whether this compound was itself bactericidal. Exposure of J2315, D1 and D3 to MC-207,110 yielded an MIC value of 640 μg/ml. In contrast, strain D4 demonstrated a MIC to MC-207,110 of 320 μg/ml, indicating that this compound exerts some antibacterial effects and that RND-4 is required at least in part for resistance to this compound. Next, the MICs of the compounds previously used to determine resistance profiles described above were re-assessed in the presence of 40 μg/ml of MC-207,110 by the agar plate method. This concentration was selected as it is well below the MIC value determined for each strain. Exposure of the parental strain J2315 or the mutant strains generated in this study to MC-207,100 did not alter the MIC profile for any of the strains tested. This is consistent with previous observations in *B. pseudomallei *where this compound did not increase drug sensitivity [22].

### Efflux of levofloxacin in *B. cenocepacia *J2315 and the D4 mutant

**Figure 2:**
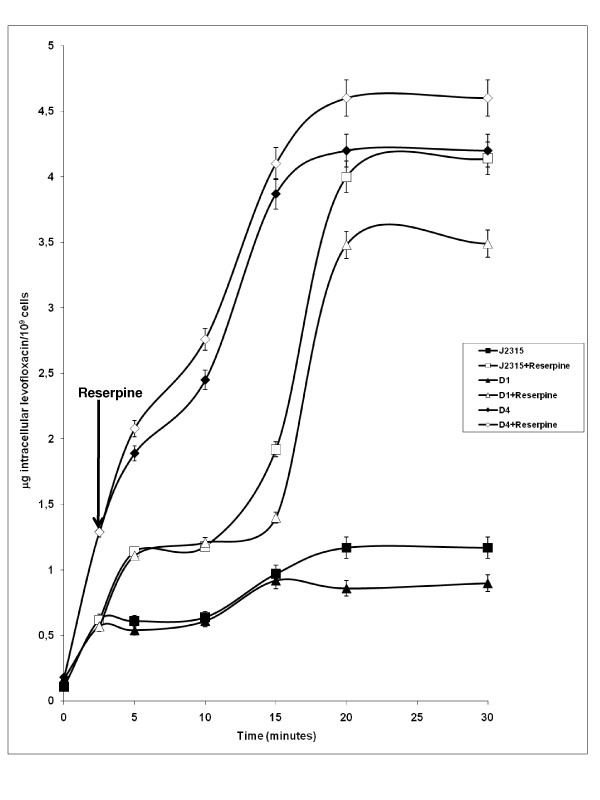
**Intracellular accumulation of levofloxacin and effect of the addition of reserpine**. Effect of the addition of reserpine on the intracellular accumulation of levofloxacin by *B. cenocepacia *J2315, D1, and D4 deleted mutants. Levofloxacin (40 μg/ml) was added to the assay mixture to initiate the assay, and reserpine (8 μg/ml) was added at the time point indicated by the arrow. Shown is the mean and standard deviation of values derived from three independent experiments.

Given that *B. cenocepacia *D4 demonstrated 8-fold reduction in its MIC for levofloxacin as compared to J2315, we determined whether the levofloxacin resistance mechanism was due to active drug efflux mediated by RND-4. This was performed by a fluorometric levofloxacin uptake assay (see Methods). Fig. 2 shows that D4 mutant bacteria rapidly accumulate levofloxacin achieving a steady-state level within 5 minutes of incubation in the presence of the drug. Levofloxacin accumulation was greatly increased (~ 80% higher) in D4 mutant bacteria as compared to the parental strain J2315. These results strongly support the notion that the RND-4 efflux pump comprised of BCAL2820, BCAL2821 and BCAL2822 functions as a levofloxacin efflux system. As a control, the uptake assay was also performed on mutant D1, which does not show any phenotype regarding the resistance profile (see Table 1). The D1 strain behaved like the wild-type strain J2315 [Fig. 2], suggesting that increased levofloxacin uptake in the mutant strains is not due to a general defect in membrane permeability.

Moreover, to determine whether the accumulation of levofloxacin was energy-dependent, reserpine was added to cells 2.5 min after the addition of levofloxacin. As shown in Fig. 2, in the presence of reserpine, in the wild-type strain and D1 mutant, the accumulation of levofloxacin increased to levels close to the values shown by D4 mutant. This demonstrates that the accumulated levels of levofloxacin were the same under de-energized conditions. This finding suggests that reserpine is able to inhibit RND-4 efflux pump, as well as the other efflux systems in J2315 and D1 strains. The addition of reserpine also increased intracellular levofloxacin accumulation in D4 mutant [Fig. 2], suggesting that additional efflux systems are expressed in the absence of this transporter, as previously reported [30].

### Evaluation of acyl homoserine lactone accumulation in the growth medium of *B. cenocepacia *J2315 and the D1, D3 and D4 mutants

**Figure 3:**
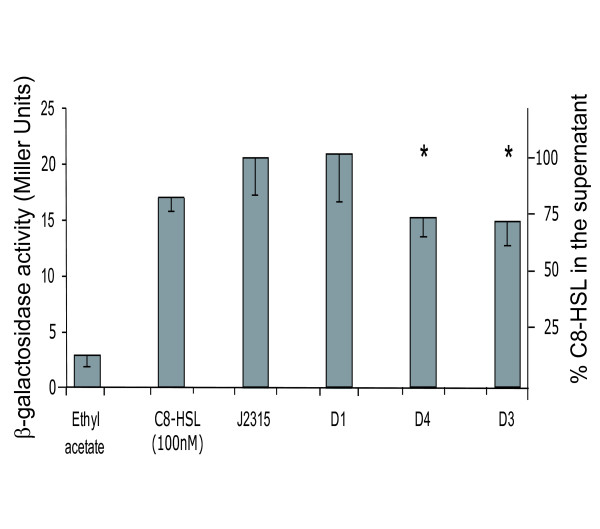
**Evaluation of AHLs accumulation in the growth medium of *B. cenocepacia *J2315 and the D1, D3, and D4 mutant strains**. C8-HSL measurement using *E. coli *(pSCR1) as described in Methods. C8-HSL was extracted from spent supernatants, AHL levels were measured with a volume of extract corresponding to 10^9 ^CFU. Values of AHL accumulated in the supernatant are expressed in Miller Units and in percentage in relation to the wild-type strain. The experiments were performed in triplicate giving comparable results. Significantly differences in AHL levels with respect J2315 are indicated by an * (ANOVA: P < 0.05; F 13.02; Dunnett's multiple Comparison test).

To determine whether the inactivated RND efflux pumps function in the transport of quorum sensing *N*-acyl homoserine lactones (AHLs) we evaluated the export of *N*-octanoyl homoserine lactone (C8-HSL). This quorum sensing molecule was previously shown to be secreted by *B. cenocepacia *[25]. Detection and quantification of C8-HSL was measured using a heterologous plasmid-based reporter assay. The plasmid pSCR1, which carries a β-galactosidase gene under the control of a C8-HSL responsive *B. cenocepacia *promoter, was transformed into *E. coli *DH5α and β-galactosidase activity determined in the presence of culture supernatants derived from control and mutant bacteria. The amount of this AHL in supernatants derived from strain D1 did not differ from the parental control. In contrast, the supernatant derived from strains D3 and D4 accumulated 30% less C8-HSL in the medium as compared to J2315 and D1 [Fig. 3]. These observations suggest that the RND transporters encoded by *BCAL1675 *and *BCAL2821 *contribute to the transport of this AHL out of *B. cenocepacia*.

## Conclusion

Employing a recently developed mutagenesis strategy [32], we successfully deleted three operons encoding RND efflux pumps in *B. cenocepacia *strain J2315. This strain is notoriously difficult to manipulate genetically, in part due to its high level of antibiotic resistance, which precludes the use of the most common selectable markers for gene exchange. The mutagenesis strategy we employed has the advantage of generating markerless deletions making it possible to repeatedly use the same antibiotic resistance cassette for subsequent gene deletions. We began our study by deleting operons encoding RND-like efflux pumps in *B. cenocepacia *J2315. The operons were selected on the basis that they are the most closely related *B. cenocepacia *efflux pumps to the Mex efflux pumps in *P. aeruginosa *[15].

Our results demonstrate that only two of the three operons targeted for deletion contribute to the antibiotic resistance of *B. cenocepacia *under the conditions tested here, and that their function contributes to the resistance of a small subset of antibiotics. Levofloxacin was one of the antibiotics to which increased sensitivity could be detected and our data indicate that RND-4 plays a role in resistance to this drug. The inability to demonstrate increased sensitivity to most classes of antibiotics supports the notion that there is functional redundancy in the efflux pumps expressed by *B. cenocepacia*. Consequently, multiple RND gene deletions in the same strain may be required to understand better their role in intrinsic antibiotic resistance. The *I-Sce*I mutagenesis system makes this possible and these experiments are currently under way in our laboratories.

Multidrug-resistance efflux pumps do not only confer antibiotic resistance, but can also function to promote colonization and persistence in the host [36]. For example, *Vibrio cholerae *RND efflux systems are required for antimicrobial resistance, optimal expression of virulence genes, and colonization of the small intestine in an infant mouse model of infection [37]. In this study, we found reduced accumulation of AHLs quorum sensing signal molecules in the growth medium of two of the RND deletion mutants. These observations suggest that these mutants have an AHL export defect that may alter quorum sensing. Importantly, it has been demonstrated that *B. cenocepacia *mutants lacking functional quorum sensing systems are attenuated in a rat model of lung infection [38]. It is likely that RND-3 and/or RND-4 might also be required for survival *in vivo *and inhibition of their function may be beneficial not only to prevent quorum sensing dependant phenomena such as biofilm formation but also to increase antibiotic sensitivity during infection.

In summary, we have demonstrated that in *B. cenocepacia*, RND efflux systems contribute to antibiotic resistance and possibly to the secretion of quorum sensing molecules. Furthermore our observations indicate that further investigation of RND efflux systems in *B. cenocepacia *is necessary to better understand how this bacterium is able to resist antibiotic treatments in the clinic and to chronically infect cystic fibrosis patients.

## Methods

### Bacterial strains and growth conditions

Bacterial strains and plasmids used in this study are listed in Table 2. Bacteria were grown in Luria-Bertani (LB) broth (Difco), with shaking at 200 rpm, or on LB agar, at 37°C. The antibiotic concentrations used were 100 μg/ml ampicillin, 50 μg/ml gentamicin, 40 μg/ml kanamycin, 50 μg/ml trimethoprim, and 12.5 μg/ml tetracycline for *E. coli*, and 800 μg/ml trimethoprim, and 300 μg/ml tetracycline for *B. cenocepacia*.

**Table 2:** Strains and plasmids

Strain or plasmid	Relevant characteristics	Source and/or reference
***B. cenocepacia *strains**		

J2315	CF clinical isolate	G. Manno
D1	J2315 Δ*BCAS0591-BCAS0593*	This study
D3	J2315 Δ*BCAL1672-BCAL1676*	This study
D4	J2315 Δ*BCAL2820-BCAL2822*	This study

***E. coli *strains**		

DH5α	F^- ^Φ80d*lacZ*Δ*M15 *Δ(*lacZYA-argF*)*U169 endA1 recA1 hsdR*17(r_K_^- ^m_K_^+^) *supE44 thi*-*1 *Δ*gyrA96 relA1*	Laboratory stock
SY327	*araD *Δ(*lac pro*) *argE*(Am) *recA56 nalA *λ *pir*; Rif^r^	[43]

**Plasmids**		

pGEM-T Easy	Vector for PCR cloning, Amp^r^	Promega
pGPI*Sce*-I	*ori*_R6K_, ΩTp^r^, *mob*^+^, containing the I*Sce*-I restriction site	[32]
pRK2013	*ori*_colE1_, RK2 derivative, Kan^r^, *mob*^+^, *tra*^+^	[44]
pDAI*Sce*-I	pDA12 encoding the I*Sce*-I homing endonuclease	[32]
pOP1/pGPI-*Sce*I	Plasmid for construction of D1 deletion mutant	This study
pOP3/pGPI-*Sce*I	Plasmid for construction of D3 deletion mutant	This study
pOP4/pGPI-*Sce*I	Plasmid for construction of D4 deletion mutant	This study
pSCR1	Amp^r^, pQF50 containing P*cepI-lacZ *and *cepR*	[42]

Amp^r^, ampicillin resistance; Kan^r^, kanamycin resistance; Rif^r^, rifampin resistance; Tet^r^, tetracycline resistance; Tp^r^, trimethoprim resistance.

### Molecular techniques

Manipulation of DNA was performed as described previously [39]. Restriction enzymes and T4 DNA ligase were purchased from GE Healthcare and used following the manufacturer's instructions. *E. coli *DH5α and *E. coli *SY327 cells were transformed by the electroporation method [39]. Plasmids were mobilized into *B. cenocepacia *J2315 by triparental mating as described previously [40], using *E. coli *DH5α carrying the helper plasmid pRK2013. Gentamicin was used to counter select against the *E. coli *donor and helper strains. All PCR reactions used the MJ Mini Personal Thermal Cycler (BioRad). To amplify PCR products *Taq *DNA polymerase, HotStar HiFidelity Polymerase kit, Hot StarTaq DNA Polymerase or Qiagen LongRange PCR kit (QIAGEN) were used and each reaction supplemented with Q solution according to the manufacturer's instructions. DNA fragments were cloned into pGEM-T Easy vector (Promega) and sequenced using the standard M13for and M13rev primers. Southern blot analyses were performed as previously described [39].

### MIC determinations

Determination of MIC (Minimal Inhibitory Concentration) for *B. cenocepacia *J2315 and the deletion mutants D1, D3, and D4 was performed by streaking 1 × 10^4 ^cells onto LB agar containing 2-fold dilutions of different drugs. The following compounds were tested to determine the resistance profile: aztreonam, ethidium bromide, chloramphenicol, gentamicin, tobramicin, nalidixic acid, ciprofloxacin, levofloxacin, norfloxacin, sparfloxacin, ampicillin, ceftazidime, erythromycin, meropenem, piperacillin, kanamycin, tetracycline, and trimethoprim. Plates were incubated at 37°C for 3 days and the growth was visually evaluated. The MIC was defined as the lowest drug concentration that prevented visible growth. The results represent the average of three independent replicas.

### Mutagenesis

The mutagenesis procedure allows for the creation of unmarked nonpolar gene deletions, as described by Flannagan *et al*. [32]. Briefly, the upstream and downstream DNA sequence that flanks (about 500 bp each) the operon targeted for deletion were cloned into pGPI*Sce*-I. This suicide plasmid contains a unique restriction site for the endonuclease I-*Sce*I. Mutagenesis plasmids were mobilized by conjugation into *B. cenocepacia *J2315 where they integrate into the chromosome by homologous recombination. Exconjugants were selected in the presence of trimethoprim (800 μg/ml) and the single crossover insertion of the mutagenic plasmid in the *B. cenocepacia *genome was confirmed by PCR analysis. Subsequently, a second plasmid, pDAI*Sce*-I (encoding the I-*Sce*I endonuclease) was introduced by conjugation. Site-specific double-strand breaks take place in the chromosome at the I-*Sce*I recognition site, resulting in tetracycline-resistant (due to the presence of pDAI-*Sce*I) and trimethoprim-susceptible (indicating the loss of the integrated mutagenic plasmid) exconjugants.

PCR amplifications of flanking regions for the construction of the mutagenesis plasmids were performed with the HotStar HiFidelity Polymerase kit (Qiagen), and the specific amplifications conditions were optimized for each primer pair, as indicated in Table 3. For the deletion of the *rnd*-1 operon, we used KO1XL- KO1BL and KO1BR-KO1KR primer pairs [Table 3]. The PCR fragments were first cloned into the pGEM-T Easy vector (Promega) and the resulting plasmids were digested with *Xba*I-*Bam*HI and *Bam*HI-*Kpn*I, respectively. The recovered fragments were cloned together into pGPI*Sce*-I digested with *Xba*I and *Kpn*I, resulting in pOP1/pGPI-*Sce*I plasmid. For the deletion of the *rnd*-3 operon, PCR amplifications of flanking regions were performed using the primer pair OP13LX-OP13LB and OP13RB-OP13RE [Table 3] and the fragments were again cloned into pGEM-T Easy. After digestion with *Xba*I-*Bam*HI and *Bam*HI-*Eco*RI, respectively, the fragments were cloned into pGPI*Sce*-I digested with *Xba*I and *Eco*RI, resulting in pOP3/pGPI-*Sce*I plasmid. For the deletion of the *rnd*-4 operon, PCR amplifications of flanking regions were performed using KO4XL-KO4NL and KO4NR-KO4KR primers [Table 3]. After cloning into pGEM-T Easy and digestion with *Xba*I-*Nde*I and *Nde*I-*Kpn*I, respectively, the fragments were cloned into pGPI*Sce*-I digested with *Xba*I and *Kpn*I, resulting in pOP4/pGPI-*Sce*I plasmid.

**Table 3:** Primers used in this work

Primer	Sequence (5'-3')	**Restriction enzyme**^a,*b*^
KO1XL^c^	GCTCTAGACTCGATGCCGCGCTGCTGGA	*Xba*I
KO1BL^c^	TTGGATCCGCGGTTCCGACTATCGCAAG	*Bam*HI
KO1BR^c^	TTGGATCCCAGGCACGCTACCGCAG	*Bam*HI
KO1KR^c^	GGGGTACCATCTGCGTCTCTTCCTTG	*Kpn*I
OP13LX^d^	GCTCTAGAGGCGGCACACCGTCGAAC	*Xba*I
OP13LB^d^	TTCGGGATCCCATCTGGCCGCCGTTTAT	*Bam*HI
OP13RB^d^	TTCGGGATCCTAATCGGCGATGTGTTGC	*Bam*HI
OP13RE^d^	TGGAATTCGCCACCCCCGCTCTCCCTTG	*Eco*RI
KO4XL^e^	GCTCTAGACCGTGATCCTGAACATCGTG	*Xba*I
KO4NL^e^	TTCATATGCGCAGACGGATCTGTACG	*Nde*I
KO4NR^e^	TTCATATGCTGCGCGACGAGGAATGC	*Nde*I
KO4KR^e^	GGGGTACCCTGCTGGTAACAATCTGTAA	*Kpn*I
KO1F	GAGGTCCAGCACGATGATG	N/A
KO1R	CGAGCATGTCCGTGACCAGT	N/A
CO13OPL	TCAAAGGGGTGTGGGCGGG	N/A
CO13OPR	GATTAAGGGAATTTCTTCTTGC	N/A
KO4F	GTCGCCGCACTTCTTCTC	N/A
KO4R	TCCTTGGTACGTCTGACC	N/A

^a ^Restriction endonuclease sites incorporated in the oligonucleotide sequences are underlined.

^b ^N/A indicates the absence of restriction site.

^c ^The PCR cycling conditions used with these primers were: 95°C for 5 min followed by 30 cycles of 94°C for 15 s, 62°C or 52°C for 1 min respectively, and 72°C for 1 min, with a final extension at 72°C for 10 min.

^d ^The PCR cycling conditions used with these primers were: 95°C for 15 min followed by 25 cycles of 94°C for 1 min, 52°C for 1 min, and 72°C for 1 min, with a final extension at 72°C for 10 min.

^e ^The PCR cycling conditions used with these primers were: 95°C for 5 min followed by 30 cycles of 94°C for 15 s, 54°C for 1 min, and 72°C for 1 min, with a final extension at 72°C for 10 min.

The mutagenesis plasmids were mobilized into *B. cenocepacia *by conjugation and mutants were selected as described above. Due to the high level of antibiotic resistance displayed by *B. cenocepacia *J2315 we used 800 μg/ml trimethoprim and 300 μg/ml tetracycline. As the trimethoprim MIC in *B. cenocepacia *J2315 is very high (256 μg/ml), we used a high concentration of antibiotic (800 μg/ml). The single crossover insertion of the mutagenic plasmid in the *B. cenocepacia *genome was confirmed by PCR. Subsequently pDAI-*Sce*I was introduced in the strain with the single crossover by conjugation. Site-specific double-strand breaks took place in the chromosome, resulting in exconjugants resistant to tetracycline and susceptible to trimethoprim. Also in this case we had to use a greater amount of tetracycline (300 μg/ml) respect to *B. cenocepacia *K56-2 (100 μg/ml), due to the high level of resistance of J2315 strain. The desired gene deletions were first confirmed by PCR amplification using primers KO1F-KO1R, CO13OPL-CO13OPR, and KO4F-KO4R for *rnd-*1, -3, and -4, respectively, and then by Southern blot hybridization of *Xho*I- (for D1 and D4 strains) or *Not*I- (for D3) cleaved genomic DNA.

### Levofloxacin accumulation assay

The accumulation of levofloxacin in *B. cenocepacia *J2315 was monitored by a fluorometric method, using the PerkinElmer LS3 fluorometer. All experiments were repeated three times. *B. cenocepacia *J2315, D1 and D4 mutant were cultured until the cells were in an exponential growth phase (OD_550 _= 0.6). The cells were then harvested by centrifugation at 4°C, washed once in 50 mM sodium phosphate buffer, pH 7.0, and resuspended in the same buffer to a final OD_550 _equal to 20. The bacterial suspension was preincubated for 10 min at 37°C in a shaking bath. Levofloxacin was added to a final concentration of 40 μg/ml. One-milliliter aliquots were collected at different time points, chilled on ice, then centrifuged at 12000 × *g *for 3 min at 4°C. The pellets were washed once with 1 ml of chilled 50 mM sodium phosphate buffer, pH 7.0 and resuspended in 1 ml of 0.1 M glycine-HCl, pH 3.0. This suspension was then lysed by incubation at room temperature for 16 h, with vigorous shaking. After 16 h, the samples were then centrifuged at 12000 × *g *for 5 min at room temperature and the fluorescence of the supernatant was measured using the excitation and emission wavelengths of 295 and 490 nm, respectively. Levofloxacin concentrations were calculated using a standard curve of the antibiotic (concentration ranging from 0.42 μg/ml to 6.38 μg/ml) in 0.1 M glycine-HCl buffer, pH 3.0. To correct for any endogenous signal the fluorescence of a control cell lysate, measured on samples not exposed to the drug, was subtracted from the experimental values. The intracellular levels of levofloxacin were expressed as drug accumulation in 10^9 ^cells, after counting of viable cells for each time point. The accumulation of levofloxacin was determined at the following time intervals: 0 min, 0 min+ drug, 2.5 min, 5 min, 10 min, 15 min, and 20 min. To determine whether levofloxacin was actively effluxed from *B. cenocepacia *J2315 and the mutant strains, reserpine (8 μg/ml) was added 2.5 min after the addition of levofloxacin and the samples were treated as described above.

### Purification, detection and quantification of N-acyl homoserine lactone (AHLs)

The purification, detection and visualization of AHL signal molecules from culture supernatants were performed as described previously [41]. Bacterial strains were inoculated in 50 ml of half diluted LB and grown at 37°C with constant agitation until OD_600 _reached 2.5. Organic extractions with ethyl acetate (0.1% acetic acid) were performed twice on each supernatant and extracts were dried and resuspended in acidified ethyl acetate in 1/1000 of the original volume. Quantification of AHLs was determined using the reporter plasmid pSCR1. This plasmid contains the *cepR *gene and the *cepI *gene promoter controlling the expression of a promoterless β-galactosidase (*lac*Z) gene and functions as a sensor of AHL molecules [42]. Overnight cultures of *E. coli *DH5α carrying pSCR1 were normalized to an OD_600 _of 0.1 in a volume of 20 ml LB containing 10 μL of the AHL purified extract (prepared as described above). 10 μL of ethyl acetate were used as negative control, while 100 nM of synthetic C8-HSL (Sigma-Fluka) was used as positive control. Cultures were then grown with agitation at 37°C for 6 h and β-galactosidase activities were determined [42].

## Authors' contributions

SBur constructed the deletion mutants, performed southern blot hybridization, MIC determination, and manuscript preparation. MRP performed levofloxacin accumulation assay. RSF designed the markerless deletion system for mutagenesis in *B. cenocepacia *and assisted with the mutagenesis experiments. SBaz performed cloning experiments. AM helped with molecular techniques. IB performed the purification, detection and quantification of acyl homoserine lactone transport. VV supervised the acyl homoserine lactone detection experiments and contributed to manuscript preparation and editing. MAV supervised the gene inactivation experiments and contributed to manuscript preparation and editing. GR designed the study and coordinated.

All authors read and approved the final manuscript.
